# Supplementary material for: Analysis of chi angle distributions in free amino acids via multiplet fitting of proton scalar couplings
Source: Magn Reson (Gott). 2024 Aug 19;5(2):103–20. doi: 10.5194/mr-5-103-2024 (PMC11570886; doi:10.5194/mr-5-103-2024)
Supplement: The supplement related to this article is available online at: https://doi.org/10.5194/mr-5-103-2024-supplement. [file mr-5-103-2024-supplement.zip › Syed2024/data/fit1d_fitnmr_output.tar.gz!/fit1d/asn/500/bmse000030_simulation_1_nmredata/1H/resonances_1d.pdf]

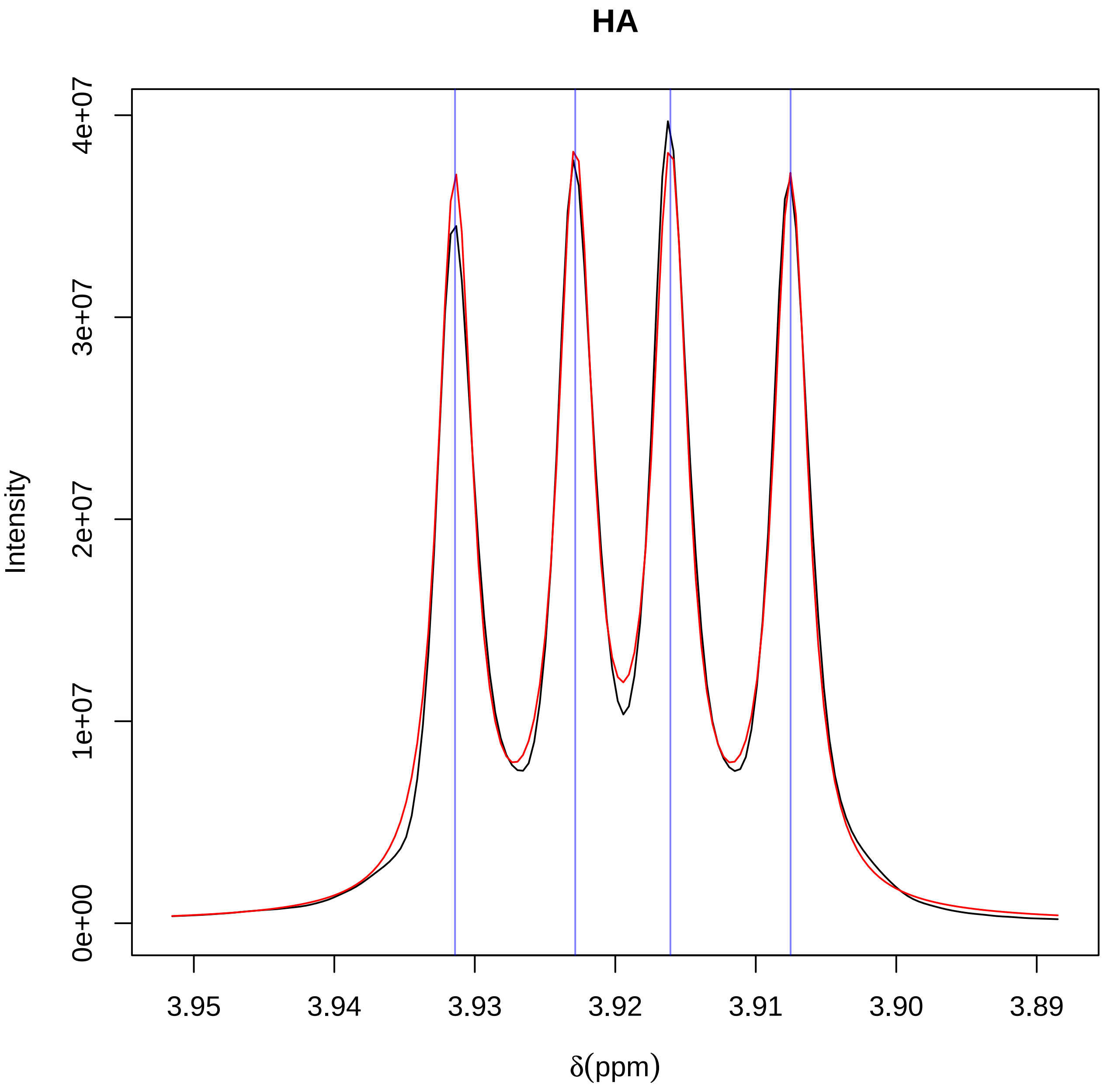

HA
Intensity
0e+00
1e+07
2e+07
3e+07
4e+07
3.95
3.94
3.93
3.92
3.91
3.90
3.89
δ(ppm)

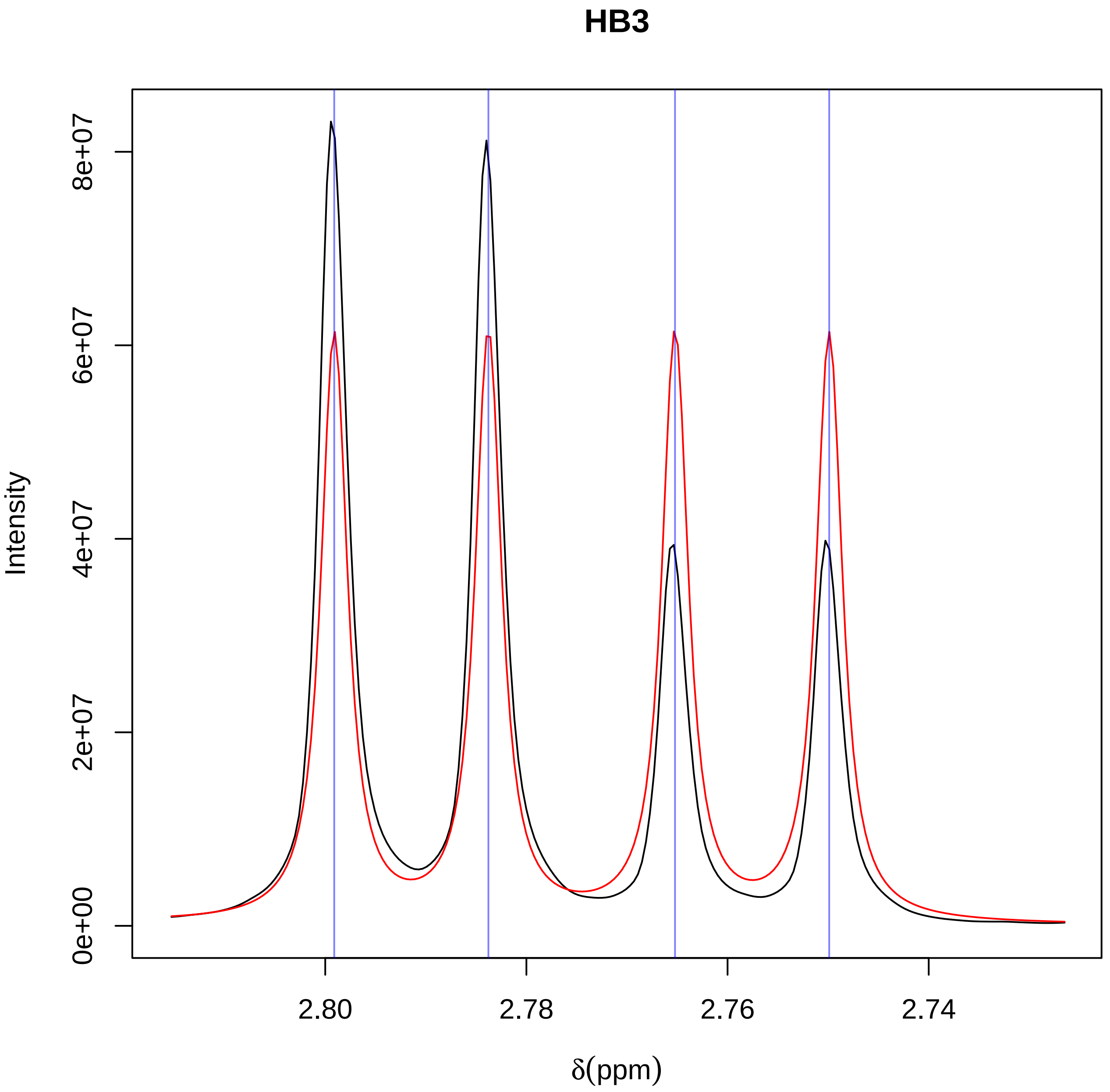

HB3
Intensity
0e+00
2e+07
4e+07
6e+07
8e+07
2.80
2.78
2.76
2.74
δ(ppm)

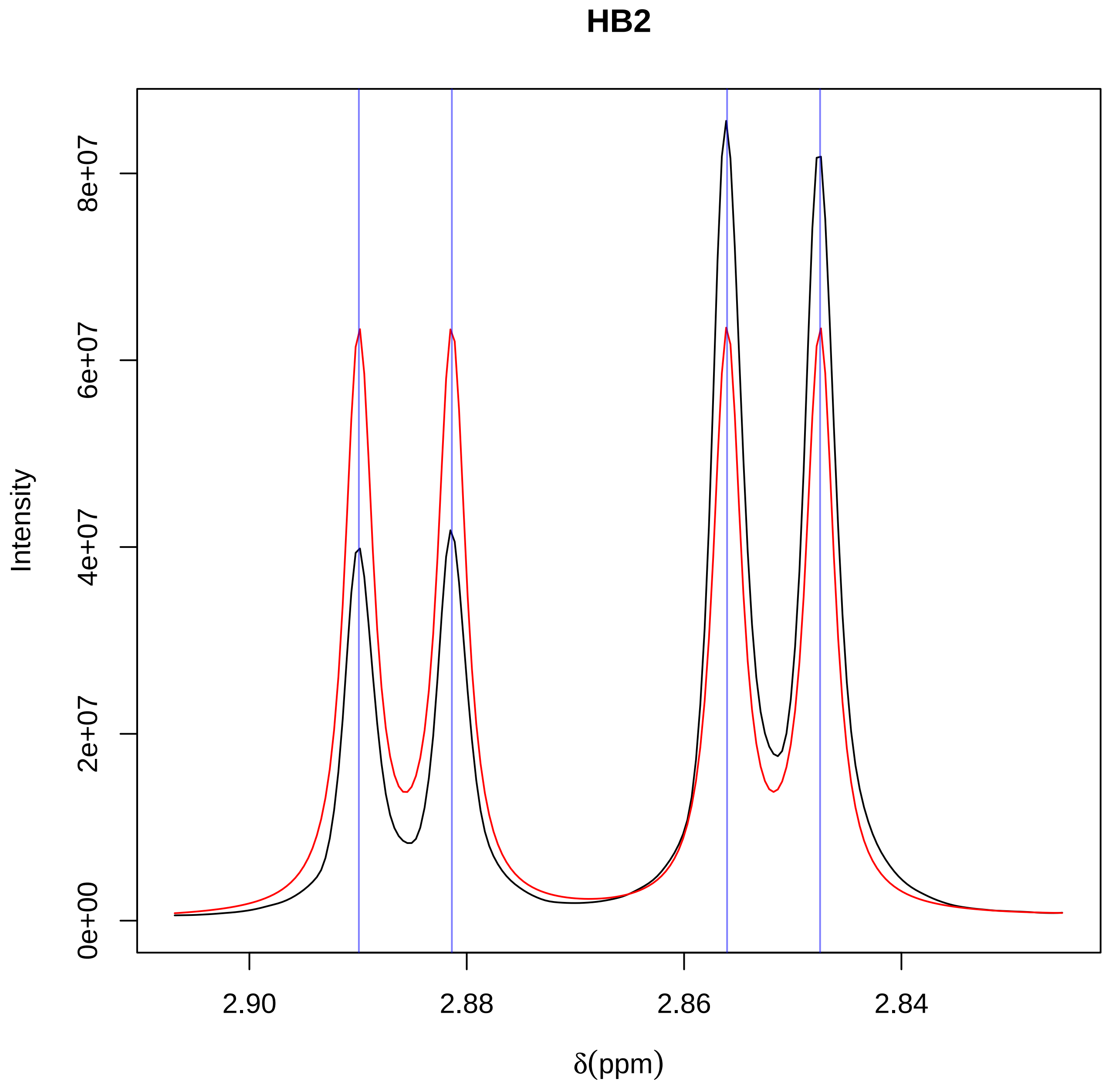
HB2
Intensity
0e+00
2e+07
4e+07
6e+07
8e+07
2.90
2.88
2.86
2.84
δ(ppm)
